# New Insights into Vaginal Environment During Pregnancy

**DOI:** 10.3389/fmolb.2021.656844

**Published:** 2021-05-17

**Authors:** Antonella Marangoni, Luca Laghi, Sara Zagonari, Giulia Patuelli, Chenglin Zhu, Claudio Foschi, Sara Morselli, Maria Federica Pedna, Vittorio Sambri

**Affiliations:** ^1^ Microbiology, DIMES, University of Bologna, Bologna, Italy; ^2^ Centre of Foodomics, Department of Agro-Food Science and Technology, University of Bologna, Cesena, Italy; ^3^ Family Advisory Health Centres, Ravenna, Italy; ^4^ Unit of Microbiology, Greater Romagna Hub Laboratory, Pievesestina, Italy

**Keywords:** vaginal microbiome, vaginal metabolome, pregnancy, miscarriage, women’s health

## Abstract

During pregnancy, the vaginal ecosystem undergoes marked changes, including a significant enrichment with *Lactobacillus* spp. and profound alterations in metabolic profiles. A deep comprehension of the vaginal environment may shed light on the physiology of pregnancy and may provide novel biomarkers to identify subjects at risk of complications (e.g., miscarriage, preterm birth). In this study, we characterized the vaginal ecosystem in Caucasian women with a normal pregnancy (n = 64) at three different gestational ages (i.e., first, second and third trimester) and in subjects (n = 10) suffering a spontaneous first trimester miscarriage. We assessed the vaginal bacterial composition (Nugent score), the vaginal metabolic profiles (^1^H-NMR spectroscopy) and the vaginal levels of two cytokines (IL-6 and IL-8). Throughout pregnancy, the vaginal microbiota became less diverse, being mainly dominated by lactobacilli. This shift was clearly associated with marked changes in the vaginal metabolome: over the weeks, a progressive reduction in the levels of dysbiosis-associated metabolites (e.g., biogenic amines, alcohols, propionate, acetate) was observed. At the same time, several metabolites, typically found in healthy vaginal conditions, reached the highest concentrations at the end of pregnancy (e.g., lactate, glycine, phenylalanine, leucine, isoleucine). Lower levels of glucose were an additional fingerprint of a normal vaginal environment. The vaginal levels of IL-6 and IL-8 were significantly associated with the number of vaginal leukocytes, as well as with the presence of vaginal symptoms, but not with a condition of dysbiosis. Moreover, IL-8 concentration seemed to be a good predictor of the presence of vaginal *Candida* spp. Cytokine concentrations were negatively correlated to lactate, serine, and glycine concentrations, whereas the levels of 4-hydroxyphenyllactate, glucose, O-acetylcholine, and choline were positively correlated with *Candida* vaginal loads. Finally, we found that most cases of spontaneous abortion were associated with an abnormal vaginal microbiome, with higher levels of selected metabolites in the vaginal environment (e.g., inosine, fumarate, xanthine, benzoate, ascorbate). No association with higher pro-inflammatory cytokines was found. In conclusion, our analysis provides new insights into the pathophysiology of pregnancy and highlights potential biomarkers to enable the diagnosis of early pregnancy loss.

## Introduction

In healthy reproductive-aged women, the vaginal microbiome is generally dominated by different species of *Lactobacillus* genus (Hickey et al., 2012; Smith and Ravel, 2017). However, the composition of the vaginal microbiome can vary in response to various factors, such as hormonal levels, sexual habits, hygiene, pregnancy, pharmaceutical treatments, and urogenital infections (Kroon et al., 2018; Noyes et al., 2018; Parolin et al., 2018).

During pregnancy, the vaginal microbiome undergoes marked changes, including a significant decrease in overall diversity, increased stability, and enrichment with *Lactobacillus* spp. (Aagaard et al., 2012; DiGiulio et al., 2015; Gupta et al., 2020).

It is well known that the vaginal bacterial composition plays a crucial role in maternal-fetal health (Fox and Eichelberger, 2015; Nelson et al., 2016). Indeed, healthy pregnancies are characterized by low numbers of vaginal bacterial communities dominated by *Lactobacillus*, whereas reduced lactobacilli with an increased bacterial diversity are associated with pregnancy-related complications and preterm birth (Prince et al., 2014; Di Simone et al., 2020).

The changes in the bacterial communities are accompanied by profound alterations in the composition of vaginal metabolites: specific vaginal molecules (e.g., acetone, ethylene glycol, formate, isopropanol, and methanol) can predict the risk of preterm birth, with a negative correlation with gestational age at birth (Ghartey et al., 2015; Ansari et al., 2020).

Moreover, several studies have investigated the association between vaginal bacterial composition and miscarriage (Zhang et al., 2019; Al-Memar et al., 2020; Fan et al., 2020; Xu et al., 2020). First trimester miscarriage seems to be associated with reduced prevalence of *Lactobacillus* spp. and higher alpha diversity compared to viable pregnancies (Al-Memar et al., 2020; Xu et al., 2020). Furthermore, it has been shown that the vaginal levels of interleukin 2 (IL-2) is higher and interleukin 10 (IL-10) lower in women with embryonic miscarriages than in control subject (Xu et al., 2020).

Despite the recent advances in the study of the vaginal environment during pregnancy (Vinturache et al., 2016; Gupta et al., 2020), the way in which a balanced vaginal microbiome helps prevent gynecological diseases and maintain maternal-fetal health remains to be fully elucidated.

Therefore, the aim of this study was to get new insights into the vaginal ecosystem in Caucasian women with a normal pregnancy (n = 64) at different gestational ages (i.e., first and second and third trimester). Ten subjects suffering a first trimester miscarriage were also included. For each woman, we characterized the vaginal bacterial composition (microscopic scoring system), the vaginal metabolic profiles (^1^H-NMR spectroscopy) and the concentrations of two cytokines (interleukin 6, IL-6, and interleukin 8, IL-8).

## Materials and Methods

### Study Group and Sample Collection

From April 2019 all the Caucasian pregnant women presenting to the Family Advisory Health Centers of Ravenna (Italy) for prenatal care were enrolled.

Exclusion criteria were the following: i) age <18 years; ii) HIV positivity; iii) body mass index (BMI) > 33; iv) medically assisted procreation; v) use of any antibiotics in the past month; vi) use of vaginal douches or topical agents in the last two weeks; vii) presence of uncontrolled chronic diseases (e.g., diabetes, autoimmune disorders, malignancies); viii) drug addiction or heavy smokers (> 15 cigarettes/day). Moreover, women with urogenital infections due to sexually transmitted pathogens (i.e., *Chlamydia trachomatis*, *Neisseria gonorrhoeae, Trichomonas vaginalis, Mycoplasma genitalium*) or erobic vaginitis were further excluded after the laboratory testing.

At gestational ages 9–13 weeks (first trimester), 20–24 weeks (second trimester), 32–34 weeks (third trimester), women underwent a clinical visit. For all patients, demographic data and information about urogenital symptoms were recorded.

Two vaginal swabs were collected at each time point (first, second trimester and third trimester). The first one (E-swab, Copan, Brescia, Italy) was used for microbiological diagnostic tests and Nugent score assessment. The second was collected with a sterile cotton bud, re-suspended in 1 ml of sterile saline, and stored at ‒80°C until use. Frozen vaginal swabs were thawed, vortexed for 1 min and removed from the liquid. The liquid was centrifuged at 10,000 × *g* for 15 min, and cell-free supernatants were used for metabolomic analysis, as described below.

A written informed consent was obtained from all subjects and the study protocol was approved by the Ethics Committee of Romagna (CEROM) (n° 2032 of 21^st^ February 2018). This study was carried out in accordance with the Declaration of Helsinki, following the recommendations of the Ethics Committee.

### Microbiological Investigations

A commercial nucleic acid amplification technique (NAAT) was used for *C. trachomatis*, *N. gonorrhoeae*, *T. vaginalis*, and *M. genitalium* detection (Seeplex STI Master Panel 1; Seegene, Seoul, KR). Microscopic examination and semi-quantitative cultures were performed for candidiasis and erobic vaginitis diagnosis (Donders et al., 2011; Yano et al., 2019). *Candida* identification at the species level was obtained by means of a matrix-assisted laser desorption/ionization time-of-flight mass spectrometry (MALDI-TOF MS) (Oliver et al., 2020).

The composition of the vaginal microbiome was assessed by a Gram stain scoring system (Nugent score), evaluating for the presence of different bacterial morphotypes (*Lactobacillus* spp., *Gardnerella vaginalis* and *Mobiluncus* spp.) (Nugent et al., 1991). Based on this score, women were divided into three groups: “H” (score 0–3; normal lactobacilli-dominated microbiota), “I” (score 4–6; intermediate microbiota), “BV” (score 7–10; bacterial vaginosis) (Zozaya-Hinchliffe et al., 2010).

Vaginal leukocytes (white blood cells: WBCs) were quantified after visualization of a minimum of five fields under light microscopy at 400×. Vaginal WBC counts were categorized into either <5 WBCs in all visualized fields (representing minimal or no inflammation) or ≥5 WBCs in at least one field visualized (considered elevated and more suggestive of significant inflammation) (Geisler et al., 2004).

### Metabolomic Analysis

Metabolomic analysis was performed by means of a ^1^H-NMR spectroscopy starting from 700 µL of the cell-free supernatants of the vaginal swabs, added to 100 μL of a D_2_O solution of 3-(trimethylsilyl)-propionic-2,2,3,3-d4 acid sodium salt (TSP) 10 mM set to pH 7.0.

^1^H-NMR spectra were recorded at 298 K with an AVANCE III spectrometer (Bruker, Milan, Italy) operating at a frequency of 600.13 MHz, equipped with Topspin software (Ver. 3.5) (Foschi et al., 2018). The signals originating from large molecules were suppressed by a CPMG filter of 400 spin-echo periods, generated by 180° pulses of 24 μs separated by 400 μs (Ventrella et al., 2016).

To each spectrum, line broadening (0.3 Hz) and phase adjustment was applied by Topspin software, while any further spectra processing, molecules quantification and data mining step was performed in R computational language (R: A Language and Environment for Statistical Computing) by means of scripts developed in house.

The spectra were aligned toward the right peak of alanine doublet, set to 1.473 ppm. The spectra were then baseline-adjusted by means of peak detection according to the “rolling ball” principle Kneen and Annegarn (1996) implemented in the “baseline” R package (Liland et al., 2010). A linear correction was then applied to each spectrum, so to make the points pertaining to the baseline randomly spread around zero.

The signals were assigned by comparing their multiplicity and chemical shift with Chenomx software data bank (ver 8.3, Chenomx Inc., Edmonton, Alberta, Canada). Quantification of the molecules was performed in the first sample acquired by employing the added TSP as an internal standard.

To compensate for differences in sample amount, any other sample was then normalized to such sample by means of probabilistic quotient normalization (Dieterle et al., 2006). Integration of the signals was performed for each molecule by means of rectangular integration.

The concentration of each molecule resulted in any case above the limit of quantification, fixed at ten times the ^1^H-NMR spectra noise (Schievano et al., 2009), assessed between the chemical shifts of 9.5 and 10.

Considering the collection method (i.e., vaginal swab) and the sample preparation, the metabolite levels should be interpreted as a concentration in the ^1^H-NMR sample and not as a primary concentration in the vaginal fluid.

### Cytokine Detection

Starting from the cell-free supernatants of the vaginal swabs, the concentration of IL-6 (pg/ml) and IL-8 (pg/ml) was determined by means of commercial ELISA assays (Simple Plex Human IL-6 and IL-8 Cartridges) run on Ella automated immunoassay system (R&D systems, Minneapolis, United States), following the manufacturer’s instructions (Brys et al., 2020).

### Data Analysis and Statistics

Data were analyzed with Prism 5.02 version for Windows (GraphPad Software, San Diego, CA, United States) and R computational language. Differences in clinical data (e.g., Nugent score) across the three stages of pregnancy were searched by Chi-square test. Differences in metabolic profiles among experimental groups were assessed by Friedman test (first vs second vs third trimester) or by Kruskal-Wallis test (H vs I vs BV), followed by Dunn’s Multiple Comparison test. Mann Whitney test was used for assessing differences in cytokines levels among the groups (e.g., presence/absence of vaginal leukocytes or *Candida* spp.) and for assessing differences between women with a normal pregnancy compared to miscarriages. Both for *Candida* presence and vaginal inflammation status, all the cases were included, irrespective of the trimester of pregnancy, also considering women with a repeated positivity across the stages of pregnancy.

Metabolite concentrations were correlated to clinical data by calculating Spearman correlation coefficient. A *p* value <0.05 was considered as statistically significant. For all metabolomic data, statistical significance was assessed after adjustment for multiple comparisons (i.e., Benjamini-Hochberg correction, with a false discovery rate of 0.25).

Trends encompassing the overall metabolome were highlighted with a robust principal component analysis (rPCA) model, according to Hubert (Hubert et al., 2005). For this purpose, we employed the PcaHubert algorithm implemented in the rrcov package of the R computational platform. The main features of the rPCA are summarized by a scoreplot and a correlation plot. The former represents the samples in the principal components (PCs) space, so that it evidences the overall structure of the data. The latter reports the correlations between the concentration of each variable and the PCs, so that it highlights the molecules mostly determining the structure of the data. Correlation between each molecule’s importance over PCs and its concentration were assessed according to Pearson.

### Data Availability Statement

Raw metabolomic data are available as a Supplementary material (Supplementary Data Sheet S1).

## Results

### Study Population

A total of 64 Caucasian pregnant women with a mean age of 31.1 ± 4.9 years (min-max: 21–44) completed the study. In addition, 10 women (mean age: 33.2 ± 7.3 years; min-max: 23–41) who had a spontaneous miscarriage at the first trimester of pregnancy (gestational age: 11–13 weeks) were also included (see specific paragraph below).

The main characteristics of the 64 women with a normal pregnancy are displayed in Table 1.

**TABLE 1:** Main characteristics of the women with a normal pregnancy (n = 64) enrolled for the study.

	1^st^ trimester	2^nd^ trimester	3^rd^ trimester	*p* value
Nugent score				
0–3 (normal microbiota)	50% (32/64)	73.4% (47/64)	79.7% (51/64)	0.0008
4–6 (intermediate microbiota)	37.5% (24/64)	15.6% (10/64)	14.1% (9/64)	
7–10 (bacterial vaginosis)	12.5% (8/64)	11% (7/64)	6.2% (4/64)	
Gestational age (weeks; mean ± SD)	10.4 ± 1.3	22.6 ± 0.8	32.5 ± 0.7	—
Vaginal *Candida* spp.	15.6% (10/64)	15.6% (10/64)	15.6% (10/64)	1.0
*-C. albicans*	6/10	6/10	8/10	
*-C. glabrata*	3/10	3/10	1/10	
*-C. krusei*	1/10	1/10	1/10	
Vaginal WBC count				
<5 (minimal or no inflammation)	76.5% (49/64)	78.1% (50/64)	73.4% (47/64)	0.8
≥5 (significant inflammation)	23.4% (15/64)	21.9% (14/64)	26.5% (17/64)	

Going from the first to the third trimester of pregnancy, we noticed a significant decrease of cases of bacterial vaginosis, together with an increase of cases characterized by a normal microbiota (*p* = 0.0008). At the first trimester (gestational age: 10.4 ± 1.3 weeks), 32 (50%) women showed a lactobacilli-dominated vaginal flora (Nugent score: 0–3), 24 (37.5%) were characterized by an intermediate microbiota (Nugent score; 4–6), whereas the remaining eight (12.5%) harbored a BV-associated microbial composition.

Conversely, at the second trimester of pregnancy (gestational age: 22.6 ± 0.8), a greater number of women showed a normal microbiota (47 women; 73.4%), with a reduction of cases of dysbiosis (15.6% intermediate microbiota; 11% BV-associated flora). Finally, at the third trimester (gestational age: 32.5 ± 0.7), most women (51; 79.7%) were characterized by a normal microbiota, with only four cases of BV (6.2%).

At the first trimester, all the women (including those with a BV-related microbiota) denied the presence of urogenital symptoms, whereas three in the second and 11 in the third reported the presence of itching and/or vaginal discharge.

Vaginal changes over time at an individual level are presented as Supplementary material (Supplementary Table S1).

A vaginal colonization/infection by *Candida* spp. was found in a total of 19 women, with 10 cases (15.6%) both at the first, second and third trimester. Four women were positive for *Candida* across all stages of pregnancy, whereas in three women *Candida* was detected in two out of the three stages. No changes in the dominant species between the three trimesters was found. *C. albicans* represented the commonest yeast found (20/30 cases), followed by *C. glabrata* (7/30) and *C. krusei* (3/30). Most cases were associated to a normal vaginal flora (17/30; 56.6%), while only three (3/30; 10%) were found in BV-positive women.

A total of 30 women (46.8%) were characterized by at least one episode of significant vaginal inflammation (≥5 WBCs in at least one field visualized) during pregnancy, with 15 cases in the first, 14 in the second, and 17 in the third trimester. Four subjects showed a high number of WBCs across all the three trimesters.

A high number of leukocytes was significantly associated with the vaginal presence of *Candida* spp. (*p* = 0.0001) but not with a status of BV (*p* = 0.57).

### IL-6 and IL-8 Detection

Overall, the mean concentration of IL-6 and IL-8 in the vaginal environment was 2.88 ± 7.6 pg/ml (min-max: 0.0–57.8) and 3,497 ± 6,299 pg/ml (min-max: 11.3 – 43,248), respectively. The levels of the two cytokines were positively correlated to each other (Spearman r: 0.63; *p* < 0.0001).

For both IL-6 and IL-8, the lowest concentrations were detected in the second trimester with a significant difference over time (IL-6, *p* = 0.02; IL-8, *p* < 0.0001).

IL-6 and IL-8 levels were significantly associated with a high number of vaginal WBCs (IL-6: 5.7 ± 12.2 vs 1.9 ± 5.2; IL-8: 7,186 ± 9,766 vs 2,334 ± 4,122; *p* < 0.0001), as well as to the presence of vaginal symptoms (IL-6: 6.03 ± 14.9 vs 2.6 ± 6.7, *p* = 0.01; IL-8: 8,552 ± 12,403 vs 3,099 ± 5,417; *p* = 0.03). Moreover, higher levels of IL-8 were significantly related to the presence of *Candida* spp. (8,583 ± 10,551 vs 2,555 ± 4,616; *p* < 0.0001) Conversely, no association between cytokine levels and Nugent score was found.

### Vaginal Metabolic Profiles

A total of 63 metabolites (mainly belonging to the groups of SCFAs, organic acids, amino acids, and biogenic amines; Supplementary Table S2) were detected and quantified by ^1^H-NMR spectroscopy.

**FIGURE 1:**
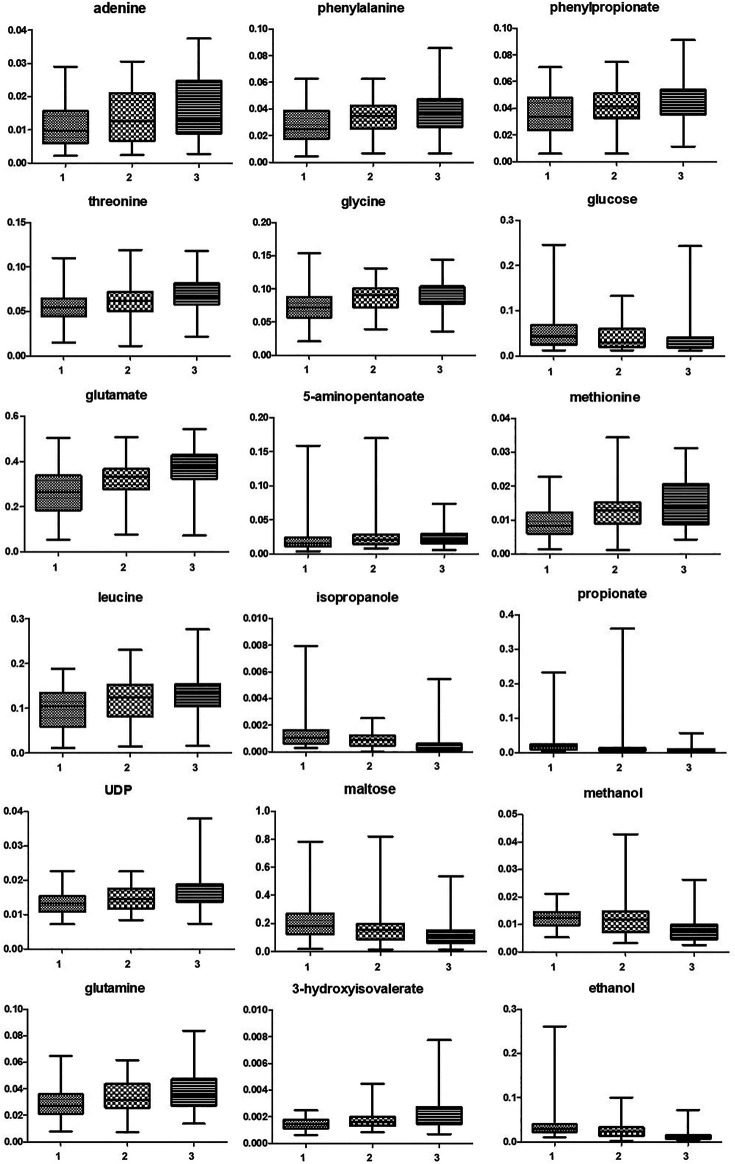
Box and whiskers representing vaginal molecules, whose concentrations showed highly significant differences (*p* < 0.0001) between the three trimesters of pregnancy. Differences were searched by Friedman test, followed by Dunn’s Multiple Comparison test. 1 = first trimester; 2 = second trimester; 3 = third trimester.

As shown in Table 2, many metabolites showed significant different concentrations going from the first to the third trimester of pregnancy. The major changes concerned the increase in the levels of lactate and of several amino acids (e.g., phenylalanine, threonine, glycine, aspartate, glutamate, isoleucine, leucine), together with the depletion of glucose, maltose, organic acids (acetate, propionate, malonate), alcohols (methanol, ethanol, isopropanol) and biogenic amines (methylamine, putrescine). Vaginal molecules, whose concentrations showed highly significant differences (*p* < 0.0001) between the three trimesters of pregnancy are shown in Figure 1.

**TABLE 2:** Molecules whose concentration (mM, mean ± SD) showed significant differences between the first, the second and the third trimester of pregnancy. Differences were searched by Friedman test, followed by Dunn’s Multiple Comparison test. A *p* value <0.05, after Benjamini-Hochberg correction, was considered as significant.

Molecules	1 trimester (mM; mean ± SD)	2 trimester (mM; mean ± SD)	3 trimester (mM; mean ± SD)	*P* value	Variation
Adenine	0.011 ± 0.006	0.014 ± 0.008	0.016 ± 0.009	<0.0001	↑ 1 vs 2; 1 vs 3; 2 vs 3
Tryptophan	0.009 ± 0.002	0.009 ± 0.002	0.01 ± 0.002	0.01	↑ 1 vs 3
Phenylalanine	0.027 ± 0.014	0.033 ± 0.014	0.038 ± 0.014	<0.0001	↑ 1vs 2; 1 vs 3
Phenylpropionate	0.035 ± 0.01	0.041 ± 0.01	0.044 ± 0.01	<0.0001	↑ 1vs 2; 1 vs 3
4-Hydroxyphenyllactate	0.0054 ± 0.002	0.0057 ± 0.002	0.0065 ± 0.002	0.0001	↑ 1 vs 3; 2 vs 3
UDP	0.013 ± 0.003	0.014 ± 0.003	0.017 ± 0.005	<0.0001	↑ 1 vs 3; 2 vs 3
Maltose	0.20 ± 0.12	0.15 ± 0.11	0.12 ± 0.09	<0.0001	↓ 1 vs 2; 1 vs 3; 2 vs 3
Hydroxyacetone	0.0020 ± 0.0008	0.0021 ± 0.001	0.0027 ± 0.001	0.008	↑ 1vs 3; 2 vs 3
Threonine	0.055 ± 0.01	0.061 ± 0.01	0.069 ± 0.01	<0.0001	↑ 1 vs 3; 2 vs 3
Lactate	2.3 ± 0.8	2.6 ± 0.8	2.6 ± 0.7	0.006	↑ 1 vs 2; 1 vs 3
*Glycine*	0.072 ± 0.026	0.087 ± 0.02	0.091 ± 0.02	<0.0001	↑ 1vs 2; 1 vs 3
Glucose	0.054 ± 0.04	0.043 ± 0.03	0.044 ± 0.04	<0.0001	↓ 1 vs 3
Methanol	0.012 ± 0.003	0.011 ± 0.006	0.008 ± 0.004	<0.0001	↓ 1vs 3; 2 vs 3
O-Acetylcholine	0.0007 ± 0.0004	0.0008 ± 0.0004	0.001 ± 0.0004	0.0005	↑ 1 vs 3; 2 vs 3
Ethanolamine	0.018 ± 0.007	0.019 ± 0.006	0.021 ± 0.008	0.0006	↑ 1 vs 3
Malonate	0.0028 ± 0.004	0.0024 ± 0.003	0.0018 ± 0.001	0.01	↓ 1 vs 3
Creatinine	0.017 ± 0.011	0.013 ± 0.009	0.011 ± 0.009	0.001	↓ 1 vs 2; 1 vs 3
Creatine	0.024 ± 0.008	0.028 ± 0.009	0.029 ± 0.01	0.0003	↑ 1 vs 2; 1 vs 3
Aspartate	0.023 ± 0.009	0.026 ± 0.008	0.028 ± 0.008	0.0005	↑ 1 vs 2; 1 vs 3
Sarcosine	0.011 ± 0.007	0.011 ± 0.006	0.013 ± 0.008	0.01	↑ 1 vs 3
Methylamine	0.002 ± 0.004	0.0012 ± 0.001	0.0012 ± 0.0007	0.002	↓ 1 vs 2
Glutamine	0.029 ± 0.01	0.034 ± 0.01	0.039 ± 0.01	<0.0001	↑ 1 vs 2; 1 vs 3
Pyruvate	0.042 ± 0.04	0.032 ± 0.04	0.021 ± 0.01	0.02	↓ 1 vs 3
Glutamate	0.26 ± 0.1	0.32 ± 0.08	0.36 ± 0.09	<0.0001	↑ 1 vs 2; 1 vs 3; 2 vs 3
4-Aminobutyrate	0.02 ± 0.03	0.019 ± 0.03	0.019 ± 0.02	0.003	↓ 1 vs 3
5-Aminopentanoate	0.025 ± 0.03	0.025 ± 0.02	0.023 ± 0.01	<0.0001	↓ 1 vs 2; 1 vs 3
Methionine	0.009 ± 0.005	0.013 ± 0.006	0.015 ± 0.007	<0.0001	↑ 1 vs 2; 1 vs 3
Proline	0.060 ± 0.005	0.056 ± 0.003	0.055 ± 0.002	0.02	↓ 1 vs 3
Acetate	0.64 ± 0.7	0.42 ± 0.5	0.30 ± 0.1	0.003	↓ 1 vs 2; 1 vs 3
Putrescine	0.008 ± 0.01	0.004 ± 0.01	0.002 ± 0.005	0.02	↓ 1 vs 2
Alanine	0.084 ± 0.04	0.087 ± 0.02	0.091 ± 0.02	0.01	↑ 1 vs 3
3-Hydroxyisovalerate	0.0014 ± 0.0004	0.0017 ± 0.0006	0.0024 ± 0.001	<0.0001	↑ 1vs 3; 2 vs 3
Ethanol	0.036 ± 0.03	0.025 ± 0.01	0.015 ± 0.01	<0.0001	↓ 1 vs 2; 1 vs 3; 2 vs 3
Isopropanol	0.001 ± 0.001	0.0009 ± 0.0006	0.0006 ± 0.001	<0.0001	↓ 1vs 3; 2 vs 3
2,3-Butanediol	0.0034 ± 0.002	0.0032 ± 0.002	0.0026 ± 0.003	0.003	↓ 1vs 3; 2 vs 3
Propionate	0.026 ± 0.03	0.021 ± 0.05	0.008 ± 0.008	<0.0001	↓ 1 vs 2; 1 vs 3
Isoleucine	0.021 ± 0.01	0.026 ± 0.01	0.027 ± 0.01	0.0001	↑ 1 vs 2; 1 vs 3
Leucine	0.10 ± 0.04	0.11 ± 0.04	0.12 ± 0.04	<0.0001	↑ 1 vs 2; 1 vs 3

These modifications were mainly driven by the composition of the vaginal microbiota. Indeed, profound differences were found in the metabolic profiles of women, stratified by the Nugent score (H vs I vs BV; Table 3).

**TABLE 3:** Concentration (mM) of vaginal metabolites determined by ^1^H-NMR, stratified by the vaginal status. Results are expressed as mean ± standard deviation. H: healthy, BV: bacterial vaginosis, I: intermediate flora. Arrows indicate significant variations (*p* < 0.05, after Benjamini-Hochberg correction) in metabolite concentration (↑ increase, ↓ decrease) between groups. Differences were searched by Kruskal-Wallis test followed by Dunn’s Multiple Comparison test.

	H (n = 130)	I (n = 43)	BV (n = 19)	*P* value	I vs H	BV vs H	BV vs I
Formate	0.040 ± 0.02	0.043 ± 0.01	0.14 ± 0.01	<0.0001		↑	↑
Adenine	0.014 ± 0.008	0.016 ± 0.007	0.007 ± 0.006	<0.0001		↓	↓
Xanthine	0.0042 ± 0.001	0.0047 ± 0.001	0.0063 ± 0.003	0.003		↑	
Tryptophan	0.01 ± 0.002	0.01 ± 0.002	0.007 ± 0.003	<0.0001		↓	↓
Benzoate	0.0031 ± 0.001	0.0036 ± 0.001	0.0038 ± 0.001	0.001	↑	↑	
Phenyalanine	0.035 ± 0.01	0.032 ± 0.01	0.018 ± 0.01	<0.0001		↓	↓
Phenylpropionate	0.043 ± 0.01	0.041 ± 0.01	0.022 ± 0.01	<0.0001		↓	↓
Tyramine	0.0038 ± 0.004	0.0034 ± 0.002	0.026 ± 0.01	<0.0001		↑	↑
Fumarate	0.0010 ± 0.0004	0.0011 ± 0.0003	0.0018 ± 0.0006	<0.0001		↑	↑
Uridine	0.008 ± 0.004	0.01 ± 0.003	0.01 ± 0.008	0.0002	↑		
Uracil	0.006 ± 0.002	0.007 ± 0.001	0.006 ± 0.001	0.003	↑		
Threonine	0.064 ± 0.01	0.06 ± 0.02	0.05 ± 0.03	0.007		↓	
Lactate	2.7 ± 0.7	2.2 ± 0.7	2.0 ± 0.8	0.0001	↓	↓	
Serine	0.07 ± 0.03	0.08 ± 0.02	0.03 ± 0.02	<0.0001		↓	↓
Glucose	0.04 ± 0.04	0.04 ± 0.03	0.06 ± 0.02	0.0002		↑	
Methanol	0.009 ± 0.004	0.01 ± 0.006	0.01 ± 0.004	<0.0001	↑	↑	
Taurine	0.07 ± 0.02	0.07 ± 0.02	0.11 ± 0.03	<0.0001		↑	↑
O-acethylcholine	0.0009 ± 0.0004	0.001 ± 0.0004	0.0004 ± 0.0005	0.0006		↓	↓
Ethanolamine	0.018 ± 0.007	0.017 ± 0.004	0.029 ± 0.01	<0.0001		↑	↑
Malonate	0.001 ± 0.0008	0.001 ± 0.0004	0.009 ± 0.006	<0.0001		↑	↑
Creatinine	0.012 ± 0.01	0.015 ± 0.01	0.019 ± 0.01	0.01		↑	
Creatine	0.026 ± 0.01	0.026 ± 0.008	0.036 ± 0.01	<0.0001		↑	↑
Cadaverine	0.008 ± 0.005	0.006 ± 0.003	0.02 ± 0.02	<0.0001		↑	↑
TMA	0.0003 ± 0.0002	0.0003 ± 0.0001	0.007 ± 0.01	<0.0001		↑	↑
Aspartate	0.027 ± 0.008	0.025 ± 0.009	0.021 ± 0.008	0.01		↓	
Sarcosine	0.013 ± 0.007	0.011 ± 0.008	0.005 ± 0.003	0.0001		↓	↓
Methylamine	0.001 ± 0.0007	0.001 ± 0.0003	0.006 ± 0.007	<0.0001		↑	↑
Succinate	0.06 ± 0.06	0.03 ± 0.03	0.3 ± 0.3	<0.0001		↑	↑
Pyruvate	0.02 ± 0.02	0.02 ± 0.02	0.1 ± 0.07	<0.0001		↑	↑
4-Aminobutyrate	0.02 ± 0.03	0.013 ± 0.006	0.01 ± 0.004	0.001		↓	
5-Aminopentanoate	0.019 ± 0.009	0.019 ± 0.008	0.071 ± 0.04	<0.0001		↑	↑
Proline	0.005 ± 0.002	0.004 ± 0.001	0.01 ± 0.007	<0.0001		↑	↑
Acetate	0.32 ± 0.2	0.28 ± 0.1	1.75 ± 1.0	<0.0001		↑	↑
Putrescine	0.001 ± 0.004	0.001 ± 0.001	0.03 ± 0.02	<0.0001		↑	↑
Butyrate	0.019 ± 0.01	0.017 ± 0.01	0.14 ± 0.2	0.0002		↑	↑
Alanine	0.08 ± 0.02	0.07 ± 0.02	0.14 ± 0.04	<0.0001		↑	↑
Ethanol	0.02 ± 0.01	0.027 ± 0.01	0.055 ± 0.055	<0.0001	↑	↑	
Isopropanol	0.0007 ± 0.0008	0.0009 ± 0.0005	0.002 ± 0.001	<0.0001	↑	↑	
2,3-Butanediol	0.002 ± 0.002	0.002 ± 0.001	0.006 ± 0.006	<0.0001		↑	↑
Propionate	0.009 ± 0.007	0.01 ± 0.007	0.09 ± 0.09	<0.0001	↑	↑	↑
Isoleucine	0.02 ± 0.009	0.02 ± 0.01	0.01 ± 0.01	<0.0001		↓	↓
Leucine	0.12 ± 0.04	0.10 ± 0.05	0.05 ± 0.03	<0.0001		↓	↓
2-Hydroxyisovalerate	0.0005 ± 0.0007	0.0005 ± 0.0003	0.006 ± 0.004	<0.0001		↑	↑

Overall, in accordance with previous investigations (Yeoman et al., 2013; Vitali et al., 2015; Ceccarani et al., 2019), BV women were characterized by higher levels of biogenic amines (tyramine, ethanolamine, cadaverine, trimethylamine -TMA-, methylamine, putrescine), alcohols, organic acids (e.g. butyrate, formate, fumarate, malonate, acetate succinate, pyruvate, propionate) and alanine, while higher levels of lactate, phenylpropionate, and diverse amino acids (e.g. tryptophan, phenyalanine, threonine, serine, isoleucine, leucine) were associated to the healthy status. Lower levels of glucose were an additional fingerprint of a normal vaginal flora.

**FIGURE 2:**
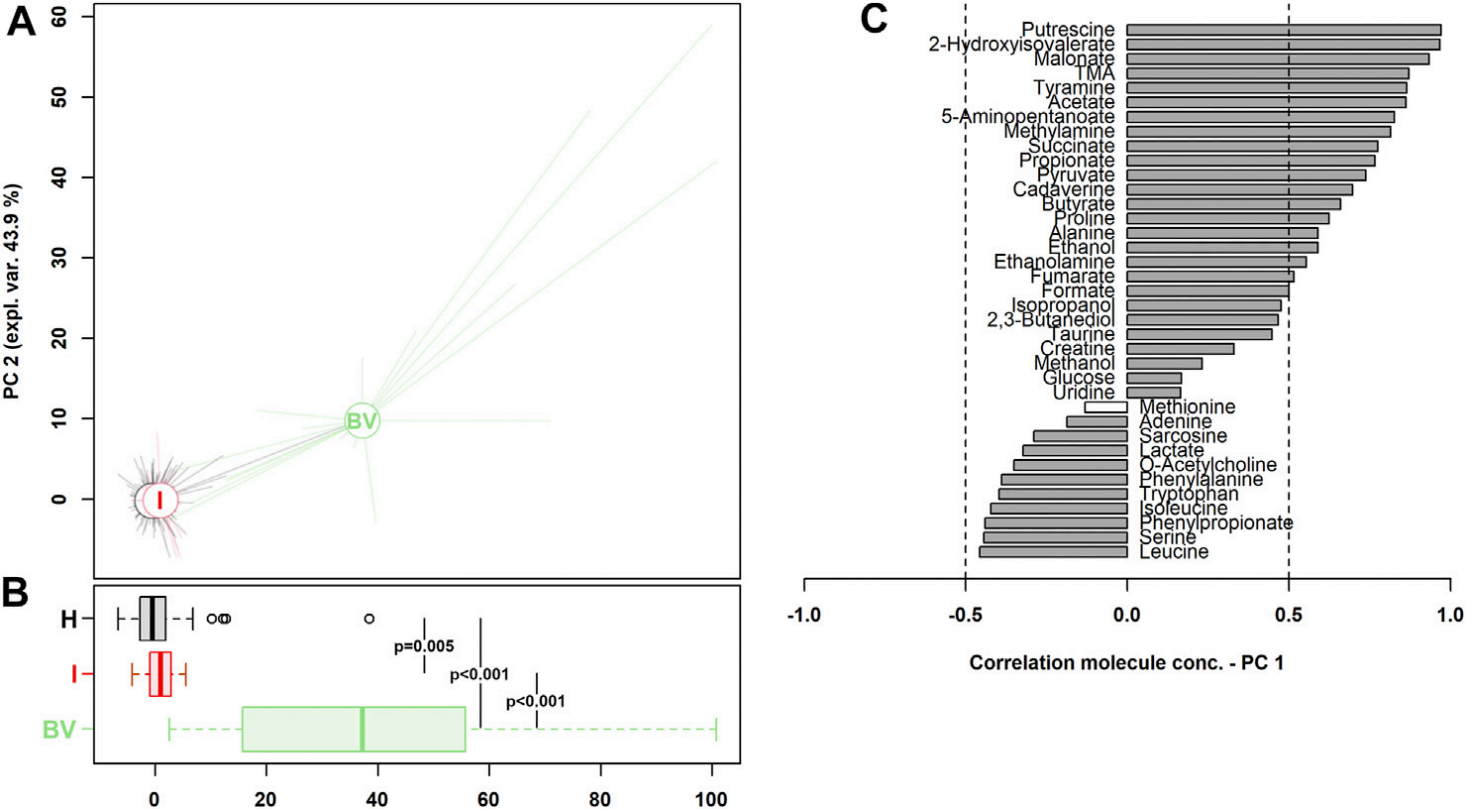
rPCA model built on the centered and scaled concentrations of the metabolites showing significant differences between groups (H vs I vs BV). **(A)** In the scoreplots, women with a healthy vaginal status (H), an intermediate flora (I) and a BV-related microbiota (BV) are represented in black, red, and green respectively, with lines connecting each subject to the median of its group. **(B)** The respective boxplots summarize the position of the groups along PC1. In the barplot **(C)**, describing the correlation between the concentration of each molecule and its importance over PC1, dark gray bars highlight statistically significant correlations (*p* < 0.05).

To obtain overviews of the differences in metabolomic profiles, a rPCA model was calculated on the basis of the most significant molecules whose concentrations were different between the groups (H vs I vs BV). All the women enrolled across the three stages of pregnancy were considered. As shown in Figure 2, a clear separation between H/I and BV metabolomes along PC1 was detected (*p* < 0.001). Nevertheless, a significant difference, even if less marked, was also noticed between H and I groups (*P* = 0.005).

As visualized in the correlation plot along PC1 (Figure 2C), higher levels of leucine, serine, phenylpropionate, isoleucine, and tryptophan were the hallmark of a healthy vaginal status during pregnancy, whereas higher concentrations of putrescine, 2-hydroxyisovalerate, malonate, TMA, tyramine, and acetate seemed to be the most significant fingerprints of BV.

Considering separately the three trimesters of pregnancy, we noticed that over time, the vaginal metabolome became less diverse and more homogeneous. In the first trimester of pregnancy (Supplementary Table S3), 36 metabolites showed significant different concentrations between healthy and BV-women, compared to 17 molecules in the second trimester (Supplementary Table S4). At the third trimester of pregnancy, only four vaginal molecules were differently concentrated between healthy and BV (Supplementary Table S5).

### Miscarriages

Most cases of spontaneous abortion were associated with an abnormal vaginal microbiome (60% intermediate flora, 20% BV condition). In these subjects, no cases of vaginal *Candida* were found, whereas two women were characterized by a high number of vaginal WBCs. For all the women, except one, it was the first miscarriage.

Differences were searched in cytokine/metabolite concentrations between women with a normal pregnancy (n = 64) and women who had a miscarriage (n = 10) at the first trimester, irrespective of the composition of the vaginal ecosystem.

**FIGURE 3:**
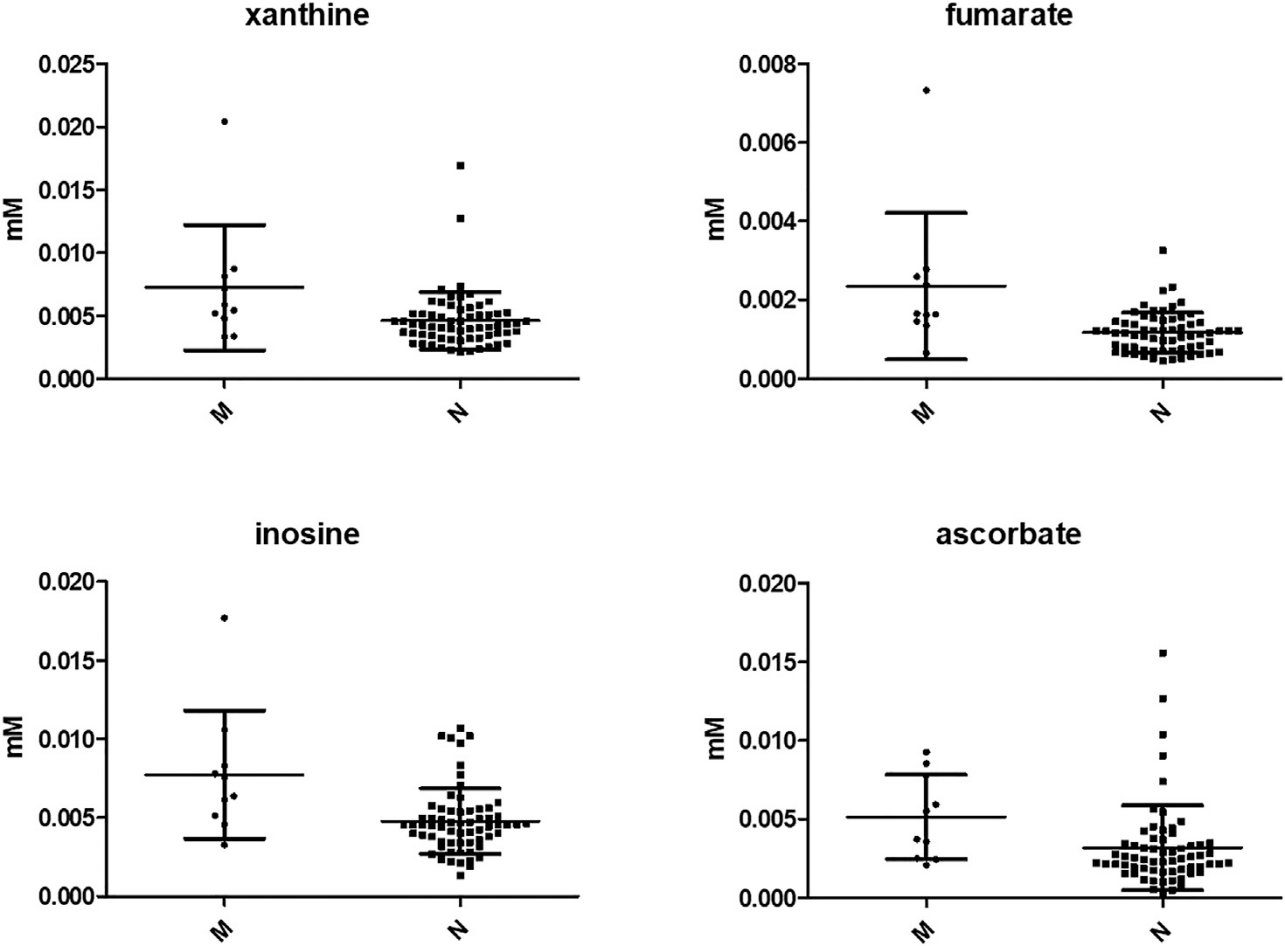
Most significant vaginal metabolites showing different concentrations between women with a normal pregnancy and subjects who had a miscarriage. N = normal pregnancy; M = miscarriage. The graphs display the mean ± SD of each metabolite. Differences were searched by Mann Whitney test.

No significant difference was observed in IL-6 and IL-8 levels between the two groups, whereas several metabolites were more concentrated in the vaginal environment of women who suffered a miscarriage (i.e., xanthine, *p* = 0.01; benzoate, *p* = 0.02, 4-hydroxyphenyllactate, *p* = 0.02; fumarate, *p* = 0.001; inosine, *p* = 0.004; UDP, *p* = 0.03; ascorbate; *p* = 0.01; ethanolamine, *p* = 0.02). The most significant are shown in Figure 3.

### Metabolome Correlations

**FIGURE 4:**
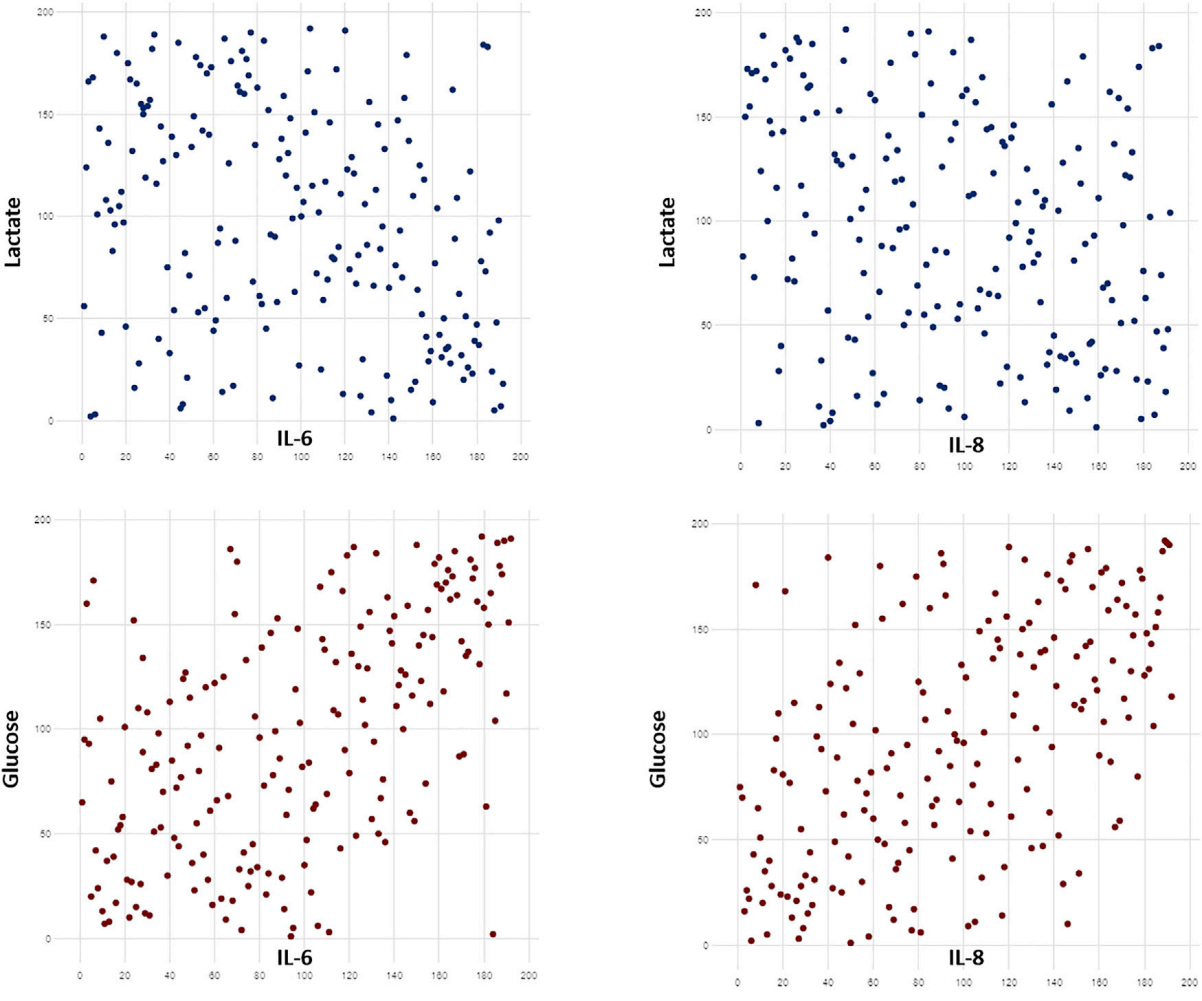
Scatterplots showing some of the most significant correlations between cytokine levels and metabolite concentration. Cytokines levels were positively correlated to glucose concentrations and negatively correlated with lactate levels (*p* < 0.0001). Raw data were transformed in ranks. Significant correlations were searched with Spearman coefficient after Benjamini-Hochberg correction.

Several correlations were found between cytokine levels and metabolite concentration (Supplementary Table S6). Among all, we detected a positive correlation between IL-6/IL-8 levels and the concentration of glucose and choline. Cytokine concentrations were negatively correlated to lactate, serine and glycine. Some of the most significant correlations are shown in Figure 4.

The levels of 4-hydroxyphenyllactate (Spearman r: 0.22; *p* = 0.002), glucose (0.18; *p* = 0.01), O-acetylcholine (0.22; *p* = 0.002) and choline (0.21; *p* = 0.004) were positively correlated with *Candida* vaginal loads.

## Discussion

A deep comprehension of the vaginal ecosystem may hold promise for unraveling the pathophysiology of pregnancy and may provide novel biomarkers to identify subjects at risk of maternal-fetal complications (i.e., miscarriage, preterm births).

In this study, we characterized the vaginal environment in 64 women with a normal pregnancy at three different gestational ages and in 10 subjects suffering a spontaneous first trimester miscarriage. In particular, we assessed the vaginal bacterial composition (Nugent score), the vaginal metabolic profiles (^1^H-NMR spectroscopy) and the concentrations of IL-6 and IL-8.

At first, we confirmed that, throughout pregnancy, the vaginal microbiota becomes less diverse, being mainly dominated by lactobacilli (Nuriel-Ohayon et al., 2016; Li et al., 2020; Rasmussen et al., 2020).

Indeed, at the third trimester of pregnancy, most women showed a normal flora (about 80%), with a few cases of BV (6.2%).

This shift toward a less complex ecosystem was clearly associated with marked changes in the vaginal metabolic profiles. Over the weeks, a progressive reduction in the levels of dysbiosis-associated metabolites (e.g., biogenic amines, alcohols, propionate, acetate) was observed. At the same time, several metabolites, typically found in healthy vaginal conditions, reached the highest concentrations at the end of pregnancy (e.g., lactate, glycine, phenylalanine, leucine, isoleucine). Moving from the first to the third trimester of pregnancy, a significant consumption of vaginal sugars (i.e., glucose and maltose) was noticed, as well.

Overall, across all stages of pregnancy, higher levels of leucine, serine, phenylpropionate, isoleucine, and tryptophan were the hallmark of a healthy vaginal status, whereas higher concentrations of putrescine, 2-hydroxyisovalerate, malonate, TMA, tyramine, and acetate were the most significant fingerprints of BV.

Most of these molecules are known to be modulated by the balance between lactobacilli and BV-related bacteria. Thus, the increase in the relative abundance of lactobacilli together with the reduction of diverse anaerobic bacteria can explain these findings.

Through carbohydrate fermentation, lactobacilli produce higher levels of lactate (Vitali et al., 2015; Ceccarani et al., 2019). Moreover, these microorganisms are known producers of branched-chain amino acids (Mutaguchi et al., 2018), being the higher concentrations of some of them, such as leucine and isoleucine, another hallmark of a vaginal healthy condition.

The reduction in several SCFAs/organic acids and biogenic amines reflect the drop of vaginal anaerobes (Vitali et al., 2015; Ceccarani et al., 2019). Malonate, acetate, and propionate are usually found at higher concentrations in the vaginal fluids of BV-positive women, being typical metabolites produced by anaerobes, as *Prevotella* and *Mobiluncus* spp. (Al-Mushrif et al., 2000). Usually, a condition of dysbiosis is also characterized by a decrease of certain protein amino acids (e.g., alanine, proline), probably due to their decarboxylation to biogenic amines (Vitali et al., 2015).

In addition, over time, the vaginal metabolic composition became less diverse and more homogeneous: indeed, in the second/third trimesters of pregnancy, women with BV showed metabolic profiles more similar to the healthy/intermediate groups, compared to the first trimester.

We can speculate that, toward the end of pregnancy, BV-affected women were characterized by a less-complex bacterial composition (i.e., less severe dysbiosis) with a reduced impact on the vaginal metabolome.

Other interesting data emerged from the detection of IL-6 and IL-8 in the vaginal environment. We found that the levels of these two cytokines were significantly associated with the number of vaginal leukocytes, as well as with the presence of vaginal symptoms. Moreover, IL-8 concentration seemed to be a good predictor of the presence of vaginal *Candida* spp.

In line with these findings, it is known that epithelial cells respond to *Candida* invasion by releasing a specific profile of cytokines, including IL-6 and IL-8, that recruit, activate, and differentiate immune cells (Verma et al., 2017).

The vaginal ecosystem of women colonized by *Candida* spp. was characterized by significantly higher levels of 4-hydroxyphenyllactate, choline, and O-acetylcholine.

Interestingly, 4-hydroxyphenyllactate has been recognized as an antifungal molecule produced by lactic acid bacteria; thus, this metabolite can certainly contribute to an anti-*Candida* effect, in synergy with other antimicrobial mechanisms (Mu et al., 2010).

*Candida* spp. produces various hydrolytic enzymes that are implicated in adhesion, invasion, and destruction of vaginal epithelial cells (Cauchie et al., 2017). Higher levels of choline can be ascribed to the production of phospholipases secreted by *Candida* during the switch from a commensal to a vaginal pathogen (Chittim et al., 2019).

Moreover, a significant association between *Candida* and glucose levels was found. High levels of glucose enhance the nutritive substrate of *Candida* and increase its adhesion, by promoting the expression of binding molecules in vaginal epithelial cells (Van Ende et al., 2019).

In line with these findings, glucose levels were positively associated with the vaginal concentrations of pro-inflammatory cytokines.

Moreover, we found a negative correlation between cytokine concentrations and vaginal lactate levels. It is worth mentioning that lactate, besides its significant antimicrobial actions, possesses immune modulating properties, thus potentially mediating anti-inflammatory effects (Aldunate et al., 2015).

We did not find any association between cytokine levels and conditions of abnormal vaginal microbiota (i.e., BV or intermediate flora). These results are in contrast with previous works showing that BV by Nugent score is associated with a significant increase in several proinflammatory cytokines/chemokines, such as IL-1α, IL-1β, IL-6, and IL-8 (Balkus et al., 2010; Kyongo et al., 2015; Florova et al., 2021).

Further studies, with a detailed evaluation of the vaginal microbiome (i.e., by means of 16 s rRNA sequencing), are needed to better understand the dynamics that take place in the vaginal ecosystem and to better relate inflammatory markers and vaginal metabolic profiles with peculiar microbial fingerprints.

When looking to women who had a first trimester miscarriage, we found that most cases of spontaneous abortion were associated with an abnormal vaginal microbiome, with higher levels of selected metabolites in the vaginal environment (e.g., fumarate, ethanolamine).

As previously observed, first trimester miscarriage can be associated with reduced prevalence of *Lactobacillus* spp. and with changes in the relative abundance of several bacterial genera, including *Fam_Finegoldia*, Lac_*Coprococcus*_3, and Lac_*Roseburia* (Al-Memar et al., 2020; Xu et al., 2020).

The increased concentration of specific vaginal metabolites could be linked to peculiar changes in the microbial composition: as an example, fumarate and ethanolamine have been recognized as BV-associated metabolites (Vitali et al., 2015).

The exact role of the vaginal metabolome in first trimester miscarriages, as well as the causative relationship between microbiota and immune responses should be further elucidated, to enable the possible diagnosis and therapeutics of early pregnancy loss.

In conclusion, our analysis described the dynamic changes of the vaginal metabolome in the different gestational ages, providing new insights into the pathophysiology of pregnancy and highlighting potential biomarkers for spontaneous abortion.

## Data Availability

The original contributions presented in the study are included in the article/Supplementary Material, further inquiries can be directed to the corresponding author.
