# Supplementary material for: New Insights into Vaginal Environment During Pregnancy
Source: Front Mol Biosci. 2021 May 17;8:656844. doi: 10.3389/fmolb.2021.656844 (PMC8165225; doi:10.3389/fmolb.2021.656844)
Supplement: Supplementary file 1 [file Table1.docx]

**Supplementary materials**

**Table S1. Vaginal changes at an individual level, stratified for the three trimesters of pregnancy.**

H: Nugent score 0-3, normal lactobacilli-dominated microbiota, ‘I’: Nugent score 4-6, intermediate microbiota; BV Nugent score 7-10, bacterial vaginosis.

| **Woman** | **1 trimester** | **2 trimester** | **3 trimester** |
| --- | --- | --- | --- |
| 1 | BV | BV | H |
| 2 | H | I | H |
| 3 | I | I | H |
| 4 | H | H | H |
| 5 | H | H | H |
| 6 | I | H | H |
| 7 | I | H | I |
| 10 | H | I | H |
| 11 | H | H | H |
| 14 | H | H | H |
| 17 | H | H | H |
| 19 | H | H | H |
| 20 | H | H | H |
| 21 | I | BV | H |
| 22 | BV | I | H |
| 25 | H | H | H |
| 26 | I | H | H |
| 27 | I | H | H |
| 28 | H | H | H |
| 29 | H | H | H |
| 30 | I | I | I |
| 31 | H | H | H |
| 32 | H | BV | H |
| 34 | H | H | I |
| 36 | I | H | H |
| 41 | I | H | H |
| 42 | I | H | H |
| 44 | I | H | H |
| 45 | I | I | I |
| 46 | BV | H | H |
| 47 | H | H | H |
| 50 | H | H | H |
| 51 | H | H | H |
| 53 | H | H | H |
| 54 | I | BV | BV |
| 55 | H | H | H |
| 56 | I | H | H |
| 57 | BV | BV | BV |
| 58 | I | H | H |
| 59 | I | H | H |
| 60 | H | H | I |
| 62 | H | H | H |
| 63 | BV | H | H |
| 64 | H | I | I |
| 65 | BV | H | H |
| 66 | I | I | H |
| 68 | I | H | I |
| 69 | I | H | BV |
| 70 | BV | H | H |
| 71 | H | H | I |
| 73 | H | H | I |
| 74 | H | BV | H |
| 75 | I | I | H |
| 76 | I | I | H |
| 78 | H | H | H |
| 79 | I | H | H |
| 81 | I | H | H |
| 82 | I | H | H |
| 83 | H | H | H |
| 93 | H | H | H |
| 96 | H | H | H |
| 100 | BV | BV | BV |
| 103 | H | H | H |
| 107 | H | H | H |

**Table S2.** **List of vaginal molecules detected and quantified by ^1^H-NMR spectroscopy.**

| **Amines** | **Organic acids** | **Amino acids** | **Alcohols** | **Sugars** | **Others** |
| --- | --- | --- | --- | --- | --- |
| Tyramine | Formate | Tryptophan | Methanol | Maltose | Hypoxanthine |
| Ethanolamine | Benzoate | Phenylalanine | Ethanol | Glucose | Adenine |
| Cadaverine | Phenylpropionate | Threonine | Isopropanol |  | Xanthine |
| TMA | 4-Hydroxyphenylacetate | Serine |  |  | Hippurate |
| DMA | 4-Hydroxyphenyllactate | Glycine |  |  | Inosine |
| Methylamine | Fumarate | Taurine |  |  | UDP |
| Putrescine | Ascorbate | Aspartate |  |  | Uridine |
|  | Lactate | Glutamate |  |  | Uracil |
|  | Malonate | Methionine |  |  | 1,3-Dihydroxyacetone |
|  | Succinate | Proline |  |  | Hydroxyacetone |
|  | Pyruvate | Alanine |  |  | sn-Glycero-3-phosphocholine |
|  | Acetate | Isoleucine |  |  | O-Acetylcholine |
|  | Butyrate | Valine |  |  | Choline |
|  | 3-Hydroxyisovalerate | Leucine |  |  | Creatinine |
|  | Propionate | Asparagine |  |  | Creatine |
|  | 2-Hydroxyisovalerate | Glutamine |  |  | Sarcosine |
|  |  |  |  |  | 4-Aminobutyrate |
|  |  |  |  |  | 5-Aminopentanoate |
|  |  |  |  |  | 2,3-Butanediol |

TMA=trimethylamine; DMA=dimethylamine

**Table S3. Concentration (mM) of vaginal metabolites determined by ^1^H-NMR at the first trimester of pregnancy, stratified by the vaginal status (H: healthy, BV: bacterial vaginosis, I: intermediate flora).** Results are expressed as mean ± standard deviation. Arrows indicate significant variations (*P* < 0.05, after Benjamini-Hochberg correction) in metabolite concentration (↑ increase, ↓ decrease) between groups. Differences were searched by Kruskal-Wallis test followed by Dunn’s Multiple Comparison test.

|  | **H (n=32)** | **I (n=24)** | **BV (n=8)** | ***P* value** | **I vs H** | **BV vs H** | **BV vs I** |
| --- | --- | --- | --- | --- | --- | --- | --- |
| Formate | 0.04 ± 0.04 | 0.04 ± 0.01 | 0.13 ± 0.13 | 0.0008 |  | ↑ |  |
| Adenine | 0.011 ± 0.006 | 0.013 ± 0.006 | 0.004 ± 0.001 | 0.0005 |  | ↓ | ↓ |
| Xanthine | 0.003 ± 0.001 | 0.004 ± 0.001 | 0.006 ± 0.004 | 0.01 |  | ↑ |  |
| Tryptophan | 0.009 ± 0.002 | 0.009 ± 0.002 | 0.006 ± 0.002 | 0.006 |  | ↓ | ↓ |
| Phenyalanine | 0.03 ± 0.01 | 0.02 ± 0.01 | 0.01 ± 0.005 | 0.005 |  | ↓ | ↓ |
| Phenylpropionate | 0.03 ± 0.01 | 0.03 ± 0.01 | 0.01 ± 0.006 | 0.001 |  | ↓ | ↓ |
| Tyramine | 0.005 ± 0.007 | 0.003 ± 0.001 | 0.03 ± 0.01 | < 0.0001 |  | ↑ | ↑ |
| Fumarate | 0.0009 ± 0.0003 | 0.001 ± 0.0003 | 0.001 ± 0.0006 | 0.0003 |  | ↑ |  |
| Uracil | 0.005 ± 0.001 | 0.007 ± 0.001 | 0.006 ± 0.001 | 0.003 | ↑ |  |  |
| Methanol | 0.010 ± 0.003 | 0.013 ± 0.003 | 0.015 ± 0.003 | 0.0007 | ↑ | ↑ |  |
| Lactate | 2.6 ± 0.7 | 2.0 ± 0.8 | 2.0 ± 1.0 | 0.005 | ↓ |  |  |
| Serine | 0.07 ± 0.04 | 0.07 ± 0.03 | 0.03 ± 0.01 | 0.006 |  | ↓ | ↓ |
| Taurine | 0.07 ± 0.02 | 0.07 ± 0.03 | 0.12 ± 0.03 | 0.004 |  | ↑ | ↑ |
| O-acethylcoline | 0.0007 ± 0.0004 | 0.0009 ± 0.0004 | 0.0002 ± 0.0003 | 0.003 |  | ↓ | ↓ |
| Ethanolamine | 0.016 ± 0.004 | 0.015 ± 0.004 | 0.032 ± 0.01 | < 0.0001 |  | ↑ | ↑ |
| Malonate | 0.001 ± 0.001 | 0.001 ± 0.0004 | 0.012 ± 0.005 | < 0.0001 |  | ↑ | ↑ |
| Creatinine | 0.01 ± 0.01 | 0.01 ± 0.01 | 0.02 ± 0.007 | 0.008 |  | ↑ | ↑ |
| Creatine | 0.02 ± 0.007 | 0.02 ± 0.007 | 0.03 ± 0.008 | 0.005 |  | ↑ | ↑ |
| Cadaverine | 0.01 ± 0.006 | 0.006 ± 0.002 | 0.04 ± 0.02 | < 0.0001 |  | ↑ | ↑ |
| TMA | 0.0004 ± 0.0003 | 0.0003 ± 0.00009 | 0.01 ± 0.009 | < 0.0001 |  | ↑ | ↑ |
| Methylamine | 0.001 ± 0.001 | 0.001 ± 0.0002 | 0.01 ± 0.008 | 0.0004 |  | ↑ | ↑ |
| DMA | 0.0009 ± 0.0003 | 0.0008 ± 0.0003 | 0.001 ± 0.0005 | 0.001 |  | ↑ | ↑ |
| Succinate | 0.07 ± 0.1 | 0.03 ± 0.03 | 0.46 ± 0.36 | 0.0001 |  | ↑ | ↑ |
| Pyruvate | 0.03 ± 0.03 | 0.02 ± 0.02 | 0.13 ± 0.03 | < 0.0001 |  | ↑ | ↑ |
| 5-Aminopentanoate | 0.015 ± 0.008 | 0.016 ± 0.006 | 0.09 ± 0.04 | < 0.0001 |  | ↑ | ↑ |
| Proline | 0.005 ± 0.003 | 0.004 ± 0.001 | 0.01 ± 0.009 | < 0.0001 |  | ↑ | ↑ |
| Acetate | 0.44 ± 0.33 | 0.32 ± 0.24 | 2.43 ± 0.6 | < 0.0001 |  | ↑ | ↑ |
| Putrescine | 0.002 ± 0.007 | 0.001 ± 0.0004 | 0.05 ± 0.02 | < 0.0001 |  | ↑ | ↑ |
| Butyrate | 0.018 ± 0.01 | 0.019 ± 0.01 | 0.22 ± 0.32 | 0.0006 |  | ↑ | ↑ |
| Alanine | 0.08 ± 0.02 | 0.06 ± 0.02 | 0.16 ± 0.02 | < 0.0001 |  | ↑ | ↑ |
| Ethanol | 0.02 ± 0.008 | 0.03 ± 0.009 | 0.08 ± 0.07 | < 0.0001 | ↑ | ↑ | ↑ |
| Isopropanol | 0.0009 ± 0.0006 | 0.001 ± 0.0004 | 0.002 ± 0.002 | < 0.0001 | ↑ | ↑ |  |
| 2,3-Butanediol | 0.002 ± 0.002 | 0.003 ± 0.001 | 0.006 ± 0.002 | 0.0002 |  | ↑ | ↑ |
| Propionate | 0.01 ± 0.01 | 0.01 ± 0.007 | 0.10 ± 0.07 | < 0.0001 |  | ↑ | ↑ |
| Isoleucine | 0.02 ± 0.01 | 0.02 ± 0.01 | 0.01 ± 0.006 | 0.004 |  | ↓ |  |
| Leucine | 0.11 ± 0.04 | 0.09 ± 0.04 | 0.05 ± 0.02 | 0.002 |  | ↓ |  |
| 2-Hydroxyisovalerate | 0.0006 ± 0.001 | 0.0006 ± 0.0003 | 0.009 ± 0.003 | < 0.0001 |  | ↑ | ↑ |
| Benzoate | 0.003 ± 0.001 | 0.003 ± 0.0008 | 0.003 ± 0.0005 | 0.008 | ↓ |  |  |
| Ascorbate | 0.002 ± 0.002 | 0.003 ± 0.002 | 0.002 ± 0.0008 | 0.009 | ↑ |  |  |
| Hydroxyacetone | 0.001 ± 0.0008 | 0.002 ± 0.0008 | 0.002 ± 0.0009 | 0.006 |  | ↑ |  |

**Table S4.** **Concentration (mM) of vaginal metabolites determined by ^1^H-NMR at the second trimester of pregnancy, stratified by the vaginal status (H: healthy, BV: bacterial vaginosis, I: intermediate flora).** Results are expressed as mean ± standard deviation. Arrows indicate significant variations (P < 0.05, after Benjamimi-Hochberg correction) in metabolite concentration (↑ increase, ↓ decrease) between groups. Differences were searched by Kruskal-Wallis test followed by Dunn’s Multiple Comparison test.

|  | **H (n=47)** | **I (n=10)** | **BV (n=7)** | ***P* value** | **I vs H** | **BV vs H** | **BV vs I** |
| --- | --- | --- | --- | --- | --- | --- | --- |
| Serine | 0.07 ± 0.02 | 0.09 ± 0.01 | 0.03 ± 0.02 | 0.0004 |  | ↓ | ↓ |
| 2-Hydroxyisovalerate | 0.0004 ± 0.0003 | 0.0003 ± 0.0002 | 0.005 ± 0.004 | 0.0002 |  | ↑ | ↑ |
| Formate | 0.03 ± 0.01 | 0.04 ± 0.01 | 0.14 ± 0.17 | 0.02 |  | ↑ |  |
| Adenine | 0.01 ± 0.008 | 0.01 ± 0.008 | 0.007 ± 0.005 | 0.003 |  | ↓ | ↓ |
| Phenylalanine | 0.03 ± 0.01 | 0.03 ± 0.01 | 0.01 ± 0.009 | 0.004 |  | ↓ | ↓ |
| Phenylpropionate | 0.04 ± 0.01 | 0.04 ± 0.01 | 0.02 ± 0.01 | 0.001 |  | ↓ | ↓ |
| Tyramine | 0.003 ± 0.002 | 0.002 ± 0.0007 | 0.02 ± 0.01 | 0.002 |  | ↑ | ↑ |
| Lactate | 2.7 ± 0.8 | 2.3 ± 0.6 | 1.8 ± 0.5 | 0.01 |  | ↓ |  |
| Malonate | 0.001 ± 0.0005 | 0.001 ± 0.0002 | 0.009 ± 0.007 | 0.006 |  | ↑ | ↑ |
| Creatine | 0.02 ± 0.008 | 0.03 ± 0.009 | 0.03 ± 0.01 | 0.009 |  | ↑ |  |
| Pyruvate | 0.02 ± 0.01 | 0.02 ± 0.01 | 0.12 ± 0.1 | 0.001 |  | ↑ |  |
| 5-Aminopentanoate | 0.02 ± 0.008 | 0.02 ± 0.008 | 0.06 ± 0.05 | 0.007 |  | ↑ |  |
| Proline | 0.004 ± 0.001 | 0.005 ± 0.002 | 0.01 ± 0.006 | 0.005 |  | ↑ | ↑ |
| Acetate | 0.28 ± 0.1 | 0.24 ± 0.08 | 1.6 ± 1 | 0.001 |  | ↑ | ↑ |
| Putrescine | 0.0009 ± 0.0004 | 0.001 ± 0.0003 | 0.03 ± 0.02 | 0.003 |  | ↑ |  |
| Isoleucine | 0.02 ± 0.009 | 0.02 ± 0.009 | 0.01 ± 0.01 | 0.01 |  | ↓ | ↓ |
| Leucine | 0.12 ± 0.04 | 0.12 ± 0.04 | 0.05 ± 0.02 | 0.001 |  | ↓ | ↓ |

**Table S5.** **Concentration (mM) of vaginal metabolites determined by ^1^H-NMR at the third trimester of pregnancy, stratified by the vaginal status (H: healthy, BV: bacterial vaginosis, I: intermediate flora).** Results are expressed as mean ± standard deviation. Arrows indicate significant variations (P < 0.05, after Benjamimi-Hochberg correction) in metabolite concentration (↑ increase, ↓ decrease) between groups. Differences were searched by Kruskal-Wallis test followed by Dunn’s Multiple Comparison test.

|  | **H (n=51)** | **I (n=9)** | **BV (n=4)** | ***P* value** | **I vs H** | **BV vs H** | **BV vs I** |
| --- | --- | --- | --- | --- | --- | --- | --- |
| Proline | 0.005 ± 0.002 | 0.005 ± 0.001 | 0.008 ± 0.001 | 0.01 |  | ↑ |  |
| Acetate | 0.29 ± 0.12 | 0.23 ± 0.07 | 0.63 ± 0.32 | 0.01 |  | ↑ | ↑ |
| Putrescine | 0.001 ± 0.002 | 0.002 ± 0.003 | 0.01 ± 0.01 | 0.01 |  | ↑ |  |
| 2-Hydroxyisovalerate | 0.0005 ± 0.0004 | 0.0004 ± 0.0001 | 0.002 ± 0.002 | 0.007 |  | ↑ | ↑ |

**Table S6. Correlations between metabolite concentration and vaginal cytokine levels (IL-6 and IL-8).** Spearman correlation coefficient was calculated. A *P* value < 0.05, after Benjamini-Hochberg correction, was considered as statistically significant.

| **Metabolites** | **IL-6** | | **IL-8** | |
| --- | --- | --- | --- | --- |
|  | **Spearman r** | ***P* value** | **Spearman r** | **P value** |
| Xanthine | 0.18 | 0.01 | - | - |
| Hypoxanthine | - | - | 0.18 | 0.01 |
| Tyramine | 0.15 | 0.03 | - | - |
| 4-Hydroxyphenyllactate | 0.23 | 0.001 | 0.31 | < 0.0001 |
| Fumarate | 0.24 | 0.001 | 0.17 | 0.02 |
| Uracil | 0.20 | 0.005 | - | - |
| Maltose | 0.28 | 0.0001 | 0.18 | 0.01 |
| Ascorbate | 0.19 | 0.008 | - | - |
| 1,3-Dihydroxyacetone | -0.15 | 0.03 | -0.24 | 0.001 |
| Hydroxyacetone | - | - | -0.17 | 0.01 |
| Lactate | -0.30 | < 0.0001 | -0.26 | < 0.0001 |
| Serine | -0.29 | < 0.0001 | -0.31 | < 0.0001 |
| Glycine | -0.26 | 0.0003 | -0.29 | < 0.0001 |
| Glucose | 0.53 | < 0.0001 | 0.54 | < 0.0001 |
| Taurine | 0.22 | 0.002 | 0.28 | 0.0001 |
| sn-Glycero-3-phosphocholine | 0.22 | 0.003 | 0.21 | 0.004 |
| Choline | 0.31 | < 0.0001 | 0.30 | < 0.0001 |
| Cadaverine | - | - | 0.19 | 0.007 |
| TMA | - | - | - | - |
| Aspartate | -0.19 | 0.008 | - | - |
| Sarcosine | -0.28 | < 0.0001 | -0.16 | 0.02 |
| DMA | -0.17 | 0.02 | - | - |
| Pyruvate | 0.21 | 0.004 | 0.38 | < 0.0001 |
| Glutamate | -0.18 | 0.01 | - | - |
| 4-Aminobutyrate | -0.24 | 0.001 | -0.16 | 0.02 |
| 5-Aminopentanoate | 0.17 | 0.01 | 0.19 | 0.007 |
| Proline | - | - | 0.16 | 0.02 |
| Putrescine | 0.18 | 0.01 | 0.15 | 0.04 |
| Butyrate | - | - | -0.15 | 0.04 |
| Alanine | - | - | 0.18 | 0.01 |
| Isoleucine | -0.20 | 0.007 | - | - |
| Leucine | -0.26 | 0.0003 | -0.18 | 0.01 |
| 2-Hydroxyisovalerate | 0.27 | 0.0002 | 0.15 | 0.03 |
